# Radioiodine therapy and Graves’ disease – Myths and reality

**DOI:** 10.1371/journal.pone.0226495

**Published:** 2020-01-13

**Authors:** Maria Teresa Plazinska, Nadia Sawicka-Gutaj, Agata Czarnywojtek, Kosma Wolinski, Małgorzata Kobylecka, Maria Karlińska, Karolina Prasek, Małgorzata Zgorzalewicz-Stachowiak, Magdalena Borowska, Paweł Gut, Marek Ruchala, Leszek Krolicki

**Affiliations:** 1 Department of Nuclear Medicine, Warsaw Medical University, Warsaw, Poland; 2 Department of Endocrinology, Metabolism and Internal Medicine, Poznan University of Medical Sciences, Poznan, Poland; 3 Department of Pharmacology, Poznan University of Medical Sciences, Poznan, Poland; 4 Department of Medical Informatics and Telemedicine, Warsaw Medical University, Warsaw, Poland; 5 Laboratory of Medical Electrodiagnostics, Department of Health Prophylaxis, Poznan University of Medical Sciences, Poznan, Poland; Consiglio Nazionale delle Ricerche, ITALY

## Abstract

**Introduction:**

Autoimmune reactions in Graves’ disease (GD) occur not only in the thyroid gland, but also in the orbital connective tissue, eyelids, extraocular muscles. The occurrence of orbitopathy in the course of GD is influenced by environmental factors, e.g. cigarette smoking.

**Objectives:**

The aim of the study was to analyze the effect of cigarette smoking on the efficacy of activity of radioiodine(131I) therapy in patients with GD. We also studied the influence of cigarette smoking and the efficacy of prednisone prophylaxis on the risk of thyroid-associated ophthalmopathy (TAO) development after radioiodine therapy (RIT) during two years of follow-up.

**Patients and methods:**

Medical records of hyperthyroid patients treated with radioiodine had been included. Patients were scheduled to visit outpatient clinics at baseline and 1, 3, 6, 9, 12, 18, and 24 months after RIT.

**Results:**

The studied group consisted of 336 patients (274 women, 62 men) diagnosed with GD and treated with RIT; 130 patients received second therapeutic dose of ^131^I due to recurrent hyperthyroidism. Among all studied patients, 220 (65.5%) were smokers and 116 (34.5%) non-smokers. In the group of smokers 115 (52.2%) of patients received single RIT, 105 (47.8%) received second dose of RAI due to recurrent hyperthyroidism. In non-smokers 91 (78.6%) received single activity of RAI, while 25 (21.4%) patients required second RIT due to recurrent hyperthyroidism. The ophthalmic symptoms in the group of smokers after RIT were less frequent, if the patient received preventative treatment in the form of oral prednisone (*P* = 0.0088).

**Conclusions:**

The results of our study suggest that cigarette smoking reduces the efficacy of treatment with ^131^I in patients with GD. The study also confirmed the effectiveness of steroid prophylaxis against TAO development or exacerbation after RIT.

## Introduction

Hyperthyroidism is found in about 2–3% of the adult population with the highest incidence in the age group of 50–59 years. Graves' disease (GD), toxic multinodular goiter, and autonomic toxic adenoma are the most common causes of these condition [1, 2]. Thyrotropin receptor autoantibodies (TSHR-Abs) plays an important in the etiology of GD [3, 4]. A special role is played by CD4 + T cells that provide the necessary assistance in the production of autoantibodies [5]. The autoimmune reaction also affects the orbital connective tissue, the eyelids, and the extraocular muscles. These pathological process can lead to the thyroid-associated orbitopathy (TAO) The pathogenesis of GD is still not fully understood. Female gender and genetic factors are the major endogenous etiological factors [4, 6–8]. Environmental factors, such as cigarette smoking, iodine deficiency and excess, stress, selenium deficiency, viral and bacterial infections, ionizing radiation, medications (interferon, estrogen), and industrial disruptors are also taken into account [9, 10]. Although the influence of cigarette smoking on TAO has been proved [7–14], the biological mechanisms responsible for the effects of smoking on the thyroid gland are not fully understood. A similar situation was observed in the case of radioidine (RAI) therapy. According to some clinical trials, TAO symptoms have worsen after RAI [11–14], especially in case of hypothyroid patients. However, some other studies does not confirm these findings [11, 15–17]. It should also be noted that 131I might augment immunologic response to factors initiating GD or could likely impair the restoration of tolerance to thyroid autoantigens [12, 15]. The pathogenesis of GD is not fully explained, which limits the therapeutic possibilities [18]. Therefore, prophylaxis against TAO development in all GD patients should be considered.

In context of such divergent reports, our aim was to analyze the effect of cigarette smoking on the efficacy of radioiodine therapy in GD patients during a two-year follow-up. Additionally, we analyzed the effect of different activity of 131I depending on the tobacco consumption. We also studied the influence of cigarette smoking and the efficacy of prednisone prophylaxis on the risk of TAO development or exacerbation after RIT.

## Materials and methods

### Patients

This was a retrospective study of medical records of patients treated in two outpatient clinics in Poland–the Department of Endocrinology, Metabolism and Internal Medicine in Poznan, and the Department of Nuclear Medicine in Warsaw. Patients were scheduled to visit outpatient clinics at baseline (before RIT), and at 1, 3, 6, 9, 12, 18, and 24 months after RIT between 2010 and 2015. Serum level of TSH, fT3 (free triiodothyronine), fT4 (free thyroxine), and TSHR-Abs were measured at every visit to the outpatient clinics. The Poznan University of Medical Sciences Ethical Committee approved this study and all participants provided informed written consent to participate in it.

### Diagnosis of GD

The diagnostic criteria of GD were as follows: presence of overt hyperthyroidism, diffuse goiter (with typical ultrasonographic and/or scintigraphic features), and positive TSHR-Abs levels either at diagnosis or at any time during the follow-up.

### Analysis of TSH, fT4, fT3, TSHR-Abs levels

The determination of the concentrations of TSH, fT4, fT3 in the blood serum was carried out using the Modular Analytics E170 analyzer (Elecsys module) from Roche Diagnostics, using the „ECLIA” electrochemiluminescence method. Estimation of TSHR-Abs concentration was performed using radioimmunoassay TRAK Human Brahms. Normal values were as follows: fT_4_−12–22 pmol/L (0,93–1,7 ng/dl), fT_3_−3.1–6.8 pmol/L (2,0–4,4 pg/ml), TSH– 0,27–4,2 μIU/ml and TSHR-Abs <1.75 IU/L.

### Clinical data before RIT

All patients had been treated with antithyroid drugs (ATD) (methimazole or propylothiouracil) for 32 ± 5 months before RIT. ATD was discontinued 5 days before the administration of RAI. Hyperthyroidism or hypothyroidism after RIT were corrected within 1 month by the therapy with ATD or L-thyroxine.

### Therapeutic regimen

The thyroid ultrasonography (The Aloka IPC-1530, Tokyo, Japan) with a 7.5-MHz linear transducer was performed and thyroid volume was calculated with the ellipsoid model (width x length x thickness x 0.52 for each lobe) [19]. A RAI uptake was measured in every patient before RIT, and 5-h and 24-h after administration of 2 MBq (54 μCi) of 131I. Activity of RAI administered to the patient are thus based upon gland size and iodine uptake using a standard formula [dose (mCi) = (μCi of 131I/g of thyroid × estimated thyroid weight)/24-h RAI uptake [18, 19]. An ablative dose (22mCi) was administered after 6 months in patients who had persistent hyperthyroidism.

### Cigarette smoking

In addition, data about cigarette smoking in patients were collected. It was assumed that habitual smokers were patients who reported that they smoked cigarettes regularly–at least one cigarette a day, during the treatment with ^131^I.

### The prophylactic use of glucocorticosteroids

In patients qualified for treatment with ^131^I who showed symptoms of mild orbitopathy, the treatment with prednisone at the dose of 0.3–0.5 mg/kg bw/a day given orally started 1–3 days after RIT, gradually reduced within 3 months [9].

### Statistical analysis

The statistical analysis was performed using the PQStat statistical package ver. 1.4.8.322 and Statistica 9.0. The comparison of TSH, fT4, fT3 and TSHR-Ab serum levels measured at different time points was performed using the Friedman test. The existence of a statistically justified relationship between cigarette smoking and the therapeutic doses of ^131^I was examined using the chi^2 test–four-pole arrays. The relationship between the administration of prednisone and the onset eye changes after RIT was analyzed using the chi^2 test for independence and Fisher's exact test. The occurrence of TAO during the two years following the treatment with RAI and the level of TSHR-Abs during this time, was analyzed using the chi^ test for independence with Yates' correction. Test probability at the level of P < 0.05 was assumed to be significant.

## Results

The study group consisted of 336 patients, including 274 women and 62 men aged 22–75 years (average 49 years) who were diagnosed with GD and treated with RIT. Clinical and biochemical characteristic at baseline are presented in Table 1. Three hundred and thirty six patients received a single therapeutic dose of ^131^I_;_ 130 of them received second therapeutic dose of ^131^I. In case of twenty-eight patients (exclusively smokers) third activity of RAI was given due to recurrent hyperthyroidism (Table 2). The ablative dose of ^131^I was administered in average 7.4 months after the treatment with the initial dose.

**Table 1:** Baseline characteristics of the smokers (n = 220) and non-smokers (n = 116) obtained at the baseline of a two-year follow-up.

Feature	Non-smokers (n = 116)	Smokers (n = 220)	*P* Value
F/M	94/22	180/40	NS
Mean age, range	36 (23–37)	33 (19–75)	NS
Duration of GTD			
< 1 year	44	79	NS
> 1 year	72	141	
TAO since (months)	4 (0–12)	7 (3–12)	NS
CAS score	2.5 (2.4 ± 1.0)	2.9 (2.6 ± 1.8)	NS
Motility score	1.0 (0–3)	1.2 (0–3)	NS
Proptosis (mm)	17.4 (17.2 ±2.0)	17.5 (17.4 ± 1.7)	NS
TSH (mU/l) (Me, Q1, Q3)	0.006 (0.005–0.08)	0.006 (0,005–0.059)	NS
free T4 (N: 11.5–21.5 pmol/L) Me, Q1-Q3	24.1 (16,65–29,9)	26.4 (17.1–32.4)	NS
Free T3 (N: 3.0–6.8 pmol/L) Me, Q1-Q3	9.3 (6.1–14.6)	9.2 (6.2–14.6)	NS
TSHRAb (N: < 2 IU/L) Me, Q1-Q3	6.6 (1.26–17.55)	7,4 (2.3–20.4)	NS

F = female, M = male, GTD = Graves’ thyroid disease, TAO = thyroid associated ophthalmopathy, CAS = clinical activity score, TSH = thyroid stimulating hormone, free T4 = l-thyroxine, T3 = tri-iodothyronine, TSHR-Ab = TSH receptor antibodies). Numerical variabies other than age are given as Me, Q1, Q3, Min., Max., Mean±SD or number (%); M, Male; F, female

**Table 2:** Radioiodine uptake (RAIU) and activity of a single therapeutic dose of ^131^I in smokers (n = 125) and non-smokers (n = 98).

Activity of ^131^I	RAIU (%) after 24 h		Number of patients (%)	
	Smokers	No smokers		
	24h	24h	Smokers 220 (64.5%)	No smokers 116 (34.5%)
8 mCi (296 MBq	45 ± 34	47 ± 22	35 (15.9%)	17 (14.7%)
10 mCi (370 MBq)	54 ± 19	61 ± 16	54 (24.5%)	32 (27.6%)
14 mCi (518 MBq)	48 ± 32	43 ± 29	0	4 (3.4%)
18mCi (666MBq)	54 ± 19	49 ± 16	124 (56.4%)	57 (49.1%)
22 mCi (814 MBq)	56 ± 22	49 ± 38	7 (3.2%)	6 (5.2%)

Among all studied patients, 220 (65.5%) were smokers, including 180 women and 40 men, 115 (52.2%) of patients received single RIT, 105 (47.8%, including 86 women and 19 men) received second dose of RAI due to recurrent hyperthyroidism. In non-smokers (n = 116, 34.5%), 91 (78.6%, including 76 women and 15 men) received single activity of RAI, while 25 (21.4%, 18 women and 7 men) patients required second RIT due to recurrent hyperthyroidism (Tab. 2). Two hundred fifty seven of the 336 studied patients (76.5%), including 37 (11%) non-smokers who had smoked in the past or whose spouse was an active smoker, were given prednisone due to mild active orbitopathy or concomitant risk factors of TAO development.

### Level of thyroid hormones (fT4, fT3) and TSH before and during the two-year follow-up

**Fig 1:**
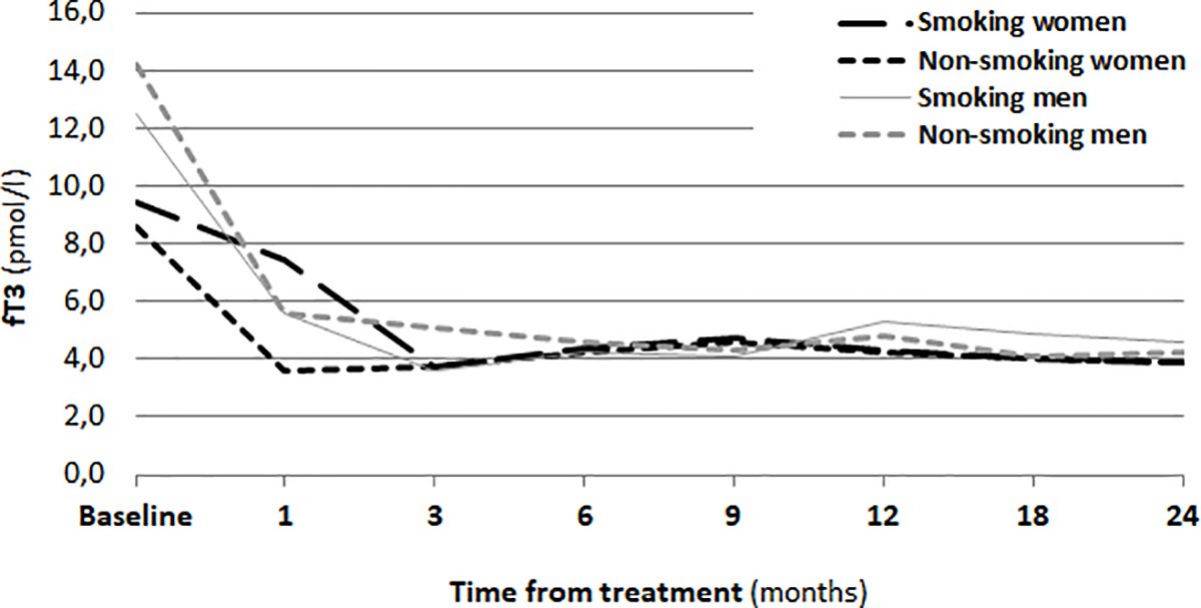
Concentration of FT3 before RIT and during a two-year follow-up.

**Fig 2:**
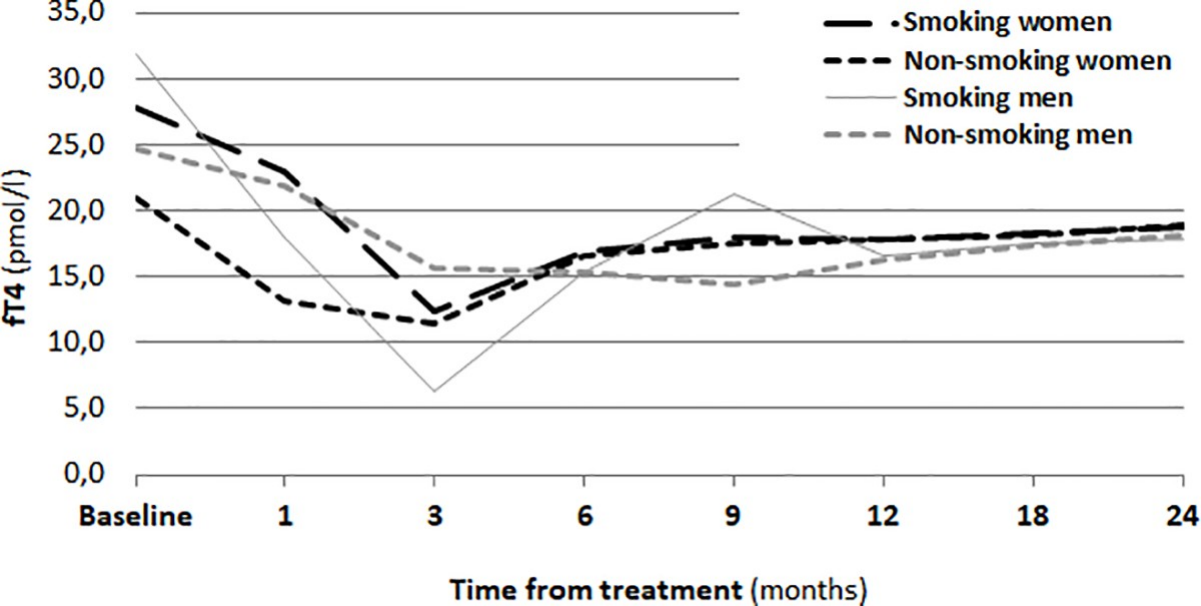
Concentration of FT4 before RIT and during a two-year follow-up.

**Fig 3:**
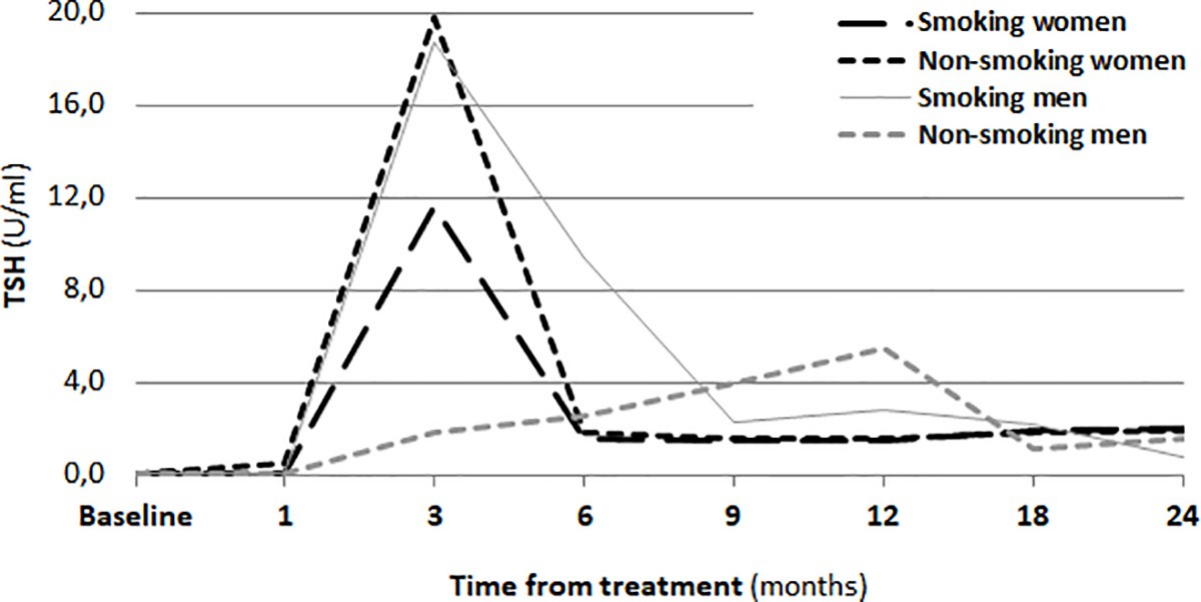
Concentration of TSH before RIT and during a two-year follow-up.

One month after RIT, a significant increase in the TSH concentration in the serum (p<0.01) was observed. The changes of concentrations of thyroid hormones (fT_3_, fT_4_) are shown in Figs 1, 2 and 3.

### TSHR-Abs before and after the RAI administration

**Fig 4:**
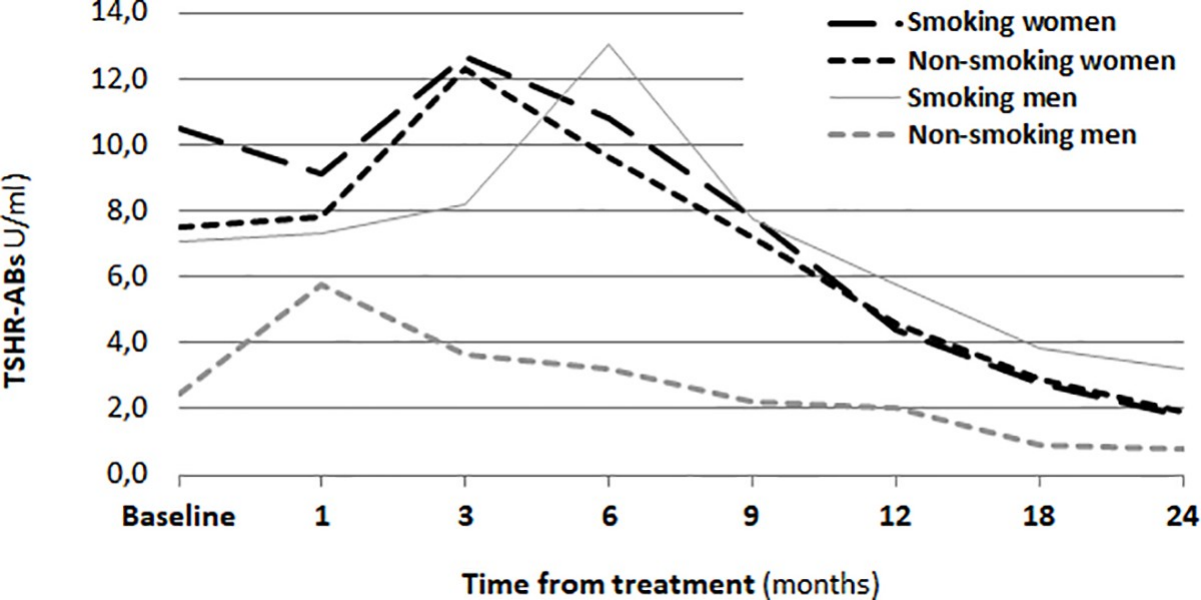
Titer of TSHR-Abs before RIT and after of a two-year follow-up.

The titer of TSHR-Abs measured 1 month after RIT increased significantly, an upward trend was observed until the third month. In the following period, after 6 and 9 months, there was a downward trend. At the subsequent time points (after one year, after 1.5 years and after 2 years) there was a significantly lower titer of TSHR-Abs than in the whole preceding period (p <0.0001) (Fig 4).

### The effect of cigarette smoking on the therapeutic activity of ^131^I

Based on the studies performed, it has been demonstrated that there is a statistically significant association (p<0.01) between cigarette smoking and the number of therapeutic doses of ^131^I taken by patients with GD. Patients who smoked cigarettes statistically more often required a second ablative dose of ^131^I.

### The average period of recurrence of hyperthyroidism after RIT

The average time of recurrence of hyperthyroidism in patients with GD was 8.58 months (min. 6 months; max 18 months; SD = 3.33 months) after the administration of the first RIT.

### The onset of TAO within 2 years after the treatment with RAI and the titer of TSHR-Abs

The relation between the onset of orbitopathy symptoms in patients with GD during a two-year of follow-up after a therapeutic dose of ^131^I and the titer of TSHR-Abs in the serum was highly significant (*P* <0.0001). In most patients who did not develop TAO the titer of THSR-Abs was within the normal range, while in the group of patients who demonstrated symptoms of orbitopathy after RIT, the initial levels of TSHR-Abs were increased (Table 3).

**Table 3:** Ocular changes in the two studied group before and after administration of RAI.

	Baseline		*P*	24 months		*P*
Ophthalmological signs	Smoking	Non-smoking		Smoking	Non-smoking	
CAS	2.9 (2.6 ± 1.8)	2.5 (2.4 ± 1.0)	NS	3.9 (3.2 ± 2.9)	2.3 (2.6 ± 1.3)	0.01
Exophthalmos (mm)	17.5 (17.4 ± 1.7)	17.4 (17.2 ±2.0)	NS	18.99 (17.5 ± 2.8)	17.9 (17.3 ±1.9)	0.05
Lid width (mm)	10.4 (10.3 ± 1.2)	10.2 (10.1 ±1.0)	NS	10.7 (10.5 ± 1.5)	10.4 (10.3 ±1.2)	NS
NOSPECS scale						
- mild	220 (100)	116 (100)	NS	110 (50)	95 (81.9)	0.01
- moderate	-	-	-	105 (47.7)	21 (18.1)	0,001
- severe	-	-	-	5 (2.3)	-	-

### The effect of prednisone prophylaxis on the onset of orbitopathy after the administration of a therapeutic dose of ^131^I

The ophthalmic symptoms in the group of smokers after RIT were much less frequent, if the patient received oral prednisone prophylaxis (*P* = 0.0088). In the group of non-smokers there was found no significant difference between occurrence of the ophthalmic symptoms after the treatment and the oral administration of prednisone (*P* = 0.08).

### The required dose of ^131^I and time to restore euthyroid state

There was observed a weak negative correlation (*P*< 0.05, R = -0.3) between the time to restore euthyroid state and the activity of the administered ^131^I. In patients with GD who were administered higher therapeutic doses of ^131^I, the time to cure was shorter than in patients who were administered lower doses of RAI.

## Discussion

Treatment with ^131^I is relatively safe and free from complications typical for surgical treatment, such as vocal cord paralysis or hypoparathyroidism. However, the thyroid damage caused by ^131^I triggers an immune response which may have serious clinical implications [20–25]. Therefore, we have analyzed, if RIT of GD is associated with rise of TSHR-Abs leading to an increased risk of TAO. We have also studied the efficacy of steroid prophylaxis against TAO progression and development.

### The effect of cigarette smoking on the amount of therapeutic doses of ^131^I

Many studies have shown that cigarette smoking has a significant effect on the thyroid gland. Tobacco smoke toxins adversely affect thyroid function, including the production of thyroid hormones, the thyroid volume and a number of autoimmune processes within the thyroid gland. It has been shown that smoking modifies levels of thyroid hormones and leads to a reduction of the thyroid stimulating hormone concentration. It has been suggested that it may also cause a slight increase in the concentration of free thyroid hormones. Smoke toxins also reduce the efficacy of therapy and increase the risk of recurrence of hyperthyroidism [12, 21, 23].

Sawicka-Gutaj N. et al. [26] studied the impact of smoking on the thyroid, taking into account the current state of medical knowledge and the latest research results. In a meta-analysis conducted by Vestergaard [27], the risk of Graves' disease was higher in patients who smoked. Studies conducted by Quadbeck et al. [28] showed much higher recurrence rate of Graves' disease in smokers in comparison to non-smokers after discontinuation of antithyroid drugs. Many studies assessed the levels of TSH, fT4 and fT3 in patients who smoked cigarettes. Analyzed studies showed that in smokers serum TSH was significantly lower than in non-smokers [29–35]. While the above mentioned studies also analyzed the recurrence risk of hypethyroidism after RIT in smoking and non-smoking patients, the effects of cigarette smoking on the amount of therapeutic doses of ^131^I taken by the patient have been not investigated yet.

According to our results, smokers with GD had significantly higher recurrence rate of hyperthyroidism after the treatment with RAI (in comparison to non-smokers).

In summary, smoking GD patients more often required repeated dose of ^131^I due to recurrent hyperthyroidism.

### The average period of recurrence of hyperthyroidism after the administration of a therapeutic dose of ^131^I

In our study, the recurrence of GD occurred in average 8.58 month after the administration of the first therapeutic dose of ^131^I. The earliest recurrence occurred 6 months after RIT and latest after18 months.

In the available literature, the average period of recurrence of hyperthyroidism after RIT in patients with GD from the moment of administration of the first therapeutic dose of ^131^I is 6 months [22, 23, 35–41]. Nwatsock J.F. *et al*. [42] demonstrated that the recurrence of hyperthyroidism after RIT occurred most frequently during the first 6 months after the administration of a therapeutic dose of ^131^I. Similar studies were conducted by Schneider et al. [37] who studied a group of 260 patients with GD for a period of 1.2 years. The recurrence of hyperthyroidism occurred in 74 patients (28.4%) and mean time to re-administration of RAI in these patients was 10.4 months.

### The onset of orbitopathy symptoms within 2 years after the treatment with RAI and the titer of TSHR- Abs in the serum

In long-term follow-up, RIT leads to a reduction of the thyroid gland volume, with subsequent reduction of the titer of TSHR-Ab [21, 43]. In the majority of GD patients without ophtalmic symptoms after RIT normal titers of TSHR-Abs had been observed, while in the majority of patients who demonstrated ophthalmic symptoms after RIT, the TSHR-Abs levels were elevated. Using NOSPECS classification, in 5 (2.3% of smokers) cases severe GO was observed, whereas in non-smokers no significant exacerbation of ophthalmopathy was observed; moderate GO was observed only in 21 cases. However, these patients turned out to be the so called passive smokers whose family members were active smokers. Ocular changes were significantly different in the two groups in CAS (*P*0.01) and exophthalmos (*P*0.05) (Table 3).

The results of current researches suggest that treatment of ^131^I can be associated with small risk of developing orbitopathy or significant worsening of already existing active TAO [22–25, 34, 35]. This phenomenon can be explained by triggered autoimmunity reflected by elevated TSHR-Abs which is caused by the release of autoantigens in the course of thyroiditis and thyroid damage induced by ^131^I [36].

According to some authors, RIT in GD patients could be the cause of TAO or its worsening in 15% of patients [44]. Lantz M. et al. [45] demonstrated that the detection of elevated levels of concentration of TSHR-Abs in the serum in patients with GD after RIT was associated with an increased risk of development of TAO symptoms.

Tobacco smoking is also a documented risk factor for the development of orbitopathy. It has been confirmed on the basis of many clinical studies that smokers or patients who smoked in the past are more likely to have symptoms of orbitopathy [10, 42, 43, 44, 45]. Bartalena et al. [46] also demonstrated that in smokers treated with ^131^I, the risk of development of orbitopathy was much higher than in non-smokers.

Research on the impact of smoking on ophthalmic symptoms was also conducted by our previous study [47]. We found a statistically significant correlation between cigarette smoking after RIT and the worsening of orbitopathy correlated with cotinine level. Therefore, these results support the significant impact of cigarette smoking on the development of TAO.

### The effect of prophylactic prednisone administration in smoking and non-smoking patients with Graves' disease before the treatment with ^131^I on the occurrence of orbitopathy

The obtained data show that ophthalmic symptoms completely disappeared or were much less severe long time after RIT (both in the group of smoking and non-smoking patients) if patients received oral prednisone. The effects of RIT presented in this paper are consistent with the data in the literature about the safety of the ^131^I treatment in patients with mild TAO [9, 22–25, 48, 49]. Prophylactic use of oral GCs prevents the progression of the ophthalmic symptoms and, which is also very important, does not affect the efficacy of RIT. However, frequent monitoring of patients, especially the group with mild orbitopathy after RIT, is important for immediate substitution therapy in case of hypothyroidism [9, 49, 50].

Prophylactic use of oral GCs in patients with mild orbitopathy treated with ^131^I is presently recommended in most specialist centers (EUGOGO guidelines 2016). However, oral GCs prophylaxis in smokers with Graves' disease committed to RIT who have not had opththalmic symptoms prior to the treatment, is debatable. However, there is much emphasis on the importance of additional risk factors which together with the applied RIT can increase the risk of orbitopathy. These factors include high titer of TSHR-Abs and the development of hypothyroidism after the treatment with ^131^I [45].

EUGOGO experts claim that in the case of smokers committed to treatment with ^131^I, one should consider preventive use of steroids, despite the absence of ophthalmic symptoms [9]. Our study showed, that 35% of patients who did not receive steroids developed the ophthalmic symptoms long time after RIT. Dederichs B. et al. [50] demonstrated that small doses of prophylactic steroids in patients who showed no signs of orbitopathy before the treatment resulted in a very small percentage (3.4%) of orbitopathy onset during the first year after RIT. In addition, there were no adverse reactions of the use of oral GCs.

Our study has some limitations. Firstly, we did not perform an analysis of the urinary level of cotinine which would be very helpful in the assessment of exacerbation of ophthalmological clinical symptoms before and after RIT, particularly in smokers. Secondly, it would be interesting to make an estimation of the haemoglobin adduct *N*-2-hydroxyethylvaline (HEV), which would be suitable to estimate the influence of smoking on the response to TAO therapy and to investigate the dependence of this response on the extent of smoking [13]

On the other hand, our publication is so interesting that it presents a very large study group during a two-year follow-up. Additionally, it clearly proves the helpful role of oral glucocorticoid therapy, especially in smokers.

## Conclusions

In conclusion, cigarette smoking decreases the efficacy of ^131^I therapy in patients with GD. It might be explained by the influence of nicotine, which constantly affects thyroid function, causing slower regression of the hyperthyroidism. This retrospective study confirmed the effectiveness of steroid prophylaxis against TAO development or exacerbation. Steroid treatment may be omitted, if there were no signs of mild orbitopathy, or if patients are not exposed to additional risk factors, such as smoking or high serum TSHR-Abs level.
